# Overexpression of hsa_circ_0136666 predicts poor prognosis and initiates osteosarcoma tumorigenesis through miR-593-3p/ZEB2 pathway

**DOI:** 10.18632/aging.103273

**Published:** 2020-05-18

**Authors:** Chao Zhang, Haibo Zhou, Kaizhen Yuan, Raoying Xie, Chun Chen

**Affiliations:** 1 Department of Orthopedics, The First Affiliated Hospital of Wenzhou Medical University, Wenzhou 325200, China; 2 Department of the Intensive Care Unit, The First Affiliated Hospital of Wenzhou Medical University, Wenzhou 325200, China; 3 Department of Radiation and Chemotherapy, The First Affiliated Hospital of Wenzhou Medical University, Wenzhou 325200, China

**Keywords:** osteosarcoma, hsa_circ_0136666, miR-593-3p, ZEB2

## Abstract

Background: Osteosarcoma (OS) is a type of malignant bone tumor with a growing incidence. Increasing studies indicate circular RNA (circRNA) has a vital function in tumorigenesis. Yet, how circRNA regulates OS development is not clear. In the present work, we aimed to investigate the roles of hsa_circ_0136666 in OS progression.

Results: hsa_circ_0136666 was shown to be upregulated in OS and correlated with advanced stage and poor prognosis. Functional investigation using CCK8, colony formation assay and Transwell assay demonstrated that hsa_circ_0136666 promoted OS proliferation, migration and invasion, but inhibited cell death. Additionally, we identified hsa_circ_0136666 was a molecular sponge for miR-593-3p to facilitate ZEB2 expression. MiR-593-3p and ZEB2 were inversely expressed in OS tissues. And hsa_circ_0136666 exerts oncogenic roles in OS relying on miR-593-3p and ZEB2.

Conclusion: Our results demonstrate the involvement of hsa_circ_0136666 in regulating OS tumorigenesis and it may be a therapeutic target.

Methods: The expression of hsa_circ_0136666 was analyzed by qRT-PCR in OS tissues and cell lines. Proliferation was measured via CCK8 and colony formation assays. Migration and invasion were determined through Transwell assay. Luciferase reporter assay was utilized to determine the interaction between hsa_circ_0136666 and miR-593-3p or between miR-593-3p and ZEB2. Animal experiment was performed to investigate the role of hsa_circ_0136666 *in vivo*.

## INTRODUCTION

Osteosarcoma (OS) is the most frequent and aggressive cancer in bone among young people [1]. OS is characterized with rapid development, metastasis and resistant to chemo-radiotherapy [2]. There is still no effective strategy to cure OS. The outcomes of OS patients were not obviously improved in the past years [3]. Therefore, it is urgently required to illustrate the molecular mechanism of OS progression and identify effective therapeutic targets for OS patients.

As a group of noncoding RNA, circular RNAs (circRNAs) are featured by a covalently closed loop and no protein-coding ability [4, 5]. As advancing of sequencing technology, more and more circRNAs have been identified in various human tissues. A growing number of studies have indicated circRNAs have important functions involved in human diseases, including cancer [6, 7]. CircRNAs have been shown to affect tumor cell proliferation, metastasis, differentiation and other biological processes [8, 9]. For example, hsa_circ_0004771 knockdown suppresses growth and promotes apoptosis of breast cancer cells [10]. CircRNA circ_0026134 modulates cell growth and migration in non-small cell lung cancer (NSCLC) by inhibiting miR-1256 and miR-1287 [11]. CircRNA circ-FOXM1 regulates NSCLC proliferation, migration and invasion via repressing miR-1304 [12]. In the recent years, the research about circRNA function in OS is merging, but limited. Therefore, it is important to uncover the regulatory functions of circRNAs in OS progression.

Hsa_circ_0136666 has been reported to promote colon cancer growth and invasiveness [13]. Additionally, upregulation of hsa_circ_0136666 causes breast cancer progression [14]. However, the role of hsa_circ_0136666 in OS is elusive. In our study, we showed that hsa_circ_0136666 expression was upregulated in OS tissues and predicted poor prognosis. And hsa_circ_0136666 loss induced suppression of proliferation, migration and invasion and increased apoptosis. Hsa_circ_0136666 was then revealed to sponge miR-593-3p and upregulate ZEB2, leading to OS progression. In sum, the present study uncovers the oncogenic role of hsa_circ_0136666 in OS and implies a potential therapeutic target.

## RESULTS

### Hsa_circ_0136666 overexpression was associated with poor prognosis in OS

**Figure 1:**
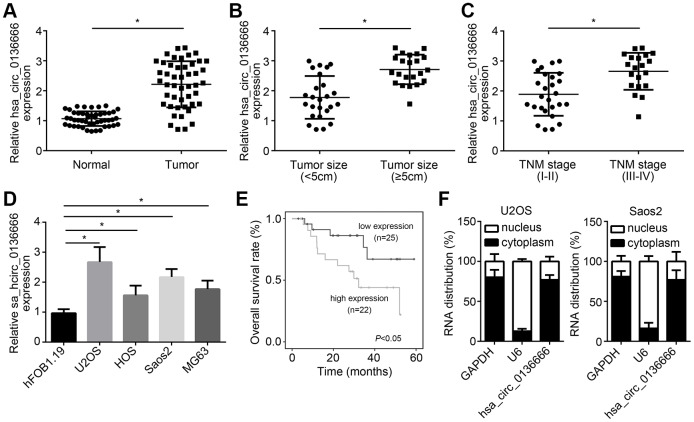
**hsa_circ_0136666 overexpression was associated with poor prognosis in OS.** (**A**) hsa_circ_0136666 was upregulated in OS tissues (n=47) compared to normal controls (n=47). (**B**) hsa_circ_0136666 expression was positively correlated with tumor size. (**C**) hsa_circ_0136666 expression was higher in advanced stages of OS tissues. (**D**) hsa_circ_0136666 expression was elevated in OS cell lines. (**E**) hsa_circ_0136666 overexpression predicted poor prognosis by Kaplan-Meier analysis. (**F**) Subcellular distribution analysis of hsa_circ_0136666 by qRT-PCR in U2OS and Saos2 cells. *P<0.05 by Student’s t-test or One-way ANOVA. Results from at least three independent experiments were presented as mean ± standard deviation.

To check the role of hsa_circ_0136666 in OS, we first measured the expression patterns of hsa_circ_0136666. Via qRT-PCR, hsa_circ_0136666 was upregulated in OS samples (Figure 1A). And hsa_circ_0136666 expression was positively correlated with tumor size and advanced stages (Figure 1B, 1C). Besides, hsa_circ_0136666 level was increased in OS cell lines compared to normal cell line (Figure 1D). Moreover, hsa_circ_0136666 overexpression is correlated with low survival rate in OS patients (Figure 1E). After qRT-PCR analysis, we found that hsa_circ_0136666 was mainly distributed in the cytoplasm of U2OS and Saos2 cells (Figure 1F).

### Knockdown of hsa_circ_0136666 inhibited proliferation, migration and invasion

**Figure 2:**
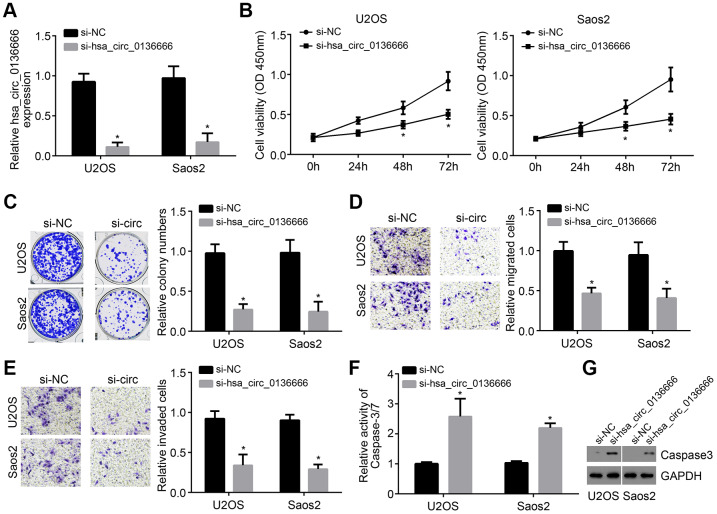
**Knockdown of hsa_circ_0136666 inhibited proliferation, migration and invasion.** (**A**) hsa_circ_0136666 expression was knocked down in U2OS and Saos2 cells in si-hsa_circ_0136666 group. (**B**, **C**) Cellular proliferation analysis using CCK8 and colony formation assay. (**D**, **E**) Determination of migration and invasion by Transwell assay (100×magnifications). (**F**) Apoptosis was tested by analysis of the Caspase-3/7 activity. (**G**) Protein level of Caspase3 was analyzed by Western blot. *P<0.05 by Student’s t-test. Results from at least three independent experiments were presented as mean ± standard deviation.

To further explore the effects of hsa_circ_0136666 on OS progression, we selected U2OS and Saos2 cells for investigation. The expression of hsa_circ_0136666 was then knocked down in U2OS and Saos2 cells by using two independent siRNAs (Figure 2A). Based on CCK8 and colony formation assay, we known hsa_circ_0136666 knockdown led to decreased proliferation of U2OS and Saos2 cells (Figure 2B, 2C). Moreover, the cell numbers of migration and invasion were also reduced after hsa_circ_0136666 silencing (Figure 2D, 2E). On the contrary, hsa_circ_0136666 silencing induced more apoptosis according to the activity of Caspase-3/7 (Figure 2F), which was confirmed by Western blotting (Figure 2G). Thus, hsa_circ_0136666 exerts oncogenic roles in OS.

### Hsa_circ_0136666 sponged miR-593-3p to upregulate ZEB2

**Figure 3:**
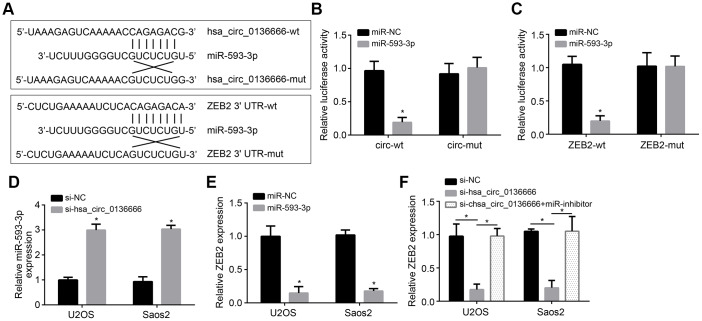
**hsa_circ_0136666 sponged miR-593-3p to upregulate ZEB2.** (**A**) Predicted binding sites between hsa_circ_0136666 and miR-593-3p or between miR-593-3p and ZEB2. (**B**, **C**) Luciferase reporter assay using hsa_circ_0136666 or ZEB2 reporter vector in U2OS cells. (**D**) Expression detection of miR-593-3p after hsa_circ_0136666 knockdown. (**E**) miR-593-3p mimics inhibited ZEB2 expression. (**F**) ZEB2 expression was inhibited after hsa_circ_0136666 knockdown. miR-inhibitor: miR-593-3p inhibitor. *P<0.05 by Student’s t-test. Results from at least three independent experiments were presented as mean ± standard deviation.

CircRNAs have been reported to be the sponge for miRNAs [6]. We observed that hsa_circ_0136666 was mainly located in the cytoplasm of U2OS cells (Figure 1F). Thus, we speculated hsa_circ_0136666 may also sponge some miRNAs. Through analysis using online tool CircInteractome, we identified miR-593-3p as a potential target of hsa_circ_0136666. And through TargetScan7 online tool, we identified ZEB2 as a potential target of miR-593-3p. To confirm it, we constructed wild-type (wt) and mutant (Mut) reporter vectors (Figure 3A). Luciferase reporter assay showed that miR-593-3p inhibited the activity of wt-hsa_circ_0136666 or wt-ZEB2 reporter (Figure 3B, 3C), demonstrating their direct interaction. Moreover, hsa_circ_0136666 knockdown promoted miR-593-3p expression while miR-593-3p suppressed ZEB2 expression (Figure 3D, 3E). And ZEB2 expression was also suppressed by hsa_circ_0136666 knockdown while reversed by miR-593-3p repression (Figure 3F). Taken together, hsa_circ_0136666 facilitates ZEB2 expression via sponging miR-593-3p.

### Effects of hsa_circ_0136666/miR-593-3p/ZEB2 axis on OS progression

**Figure 4:**
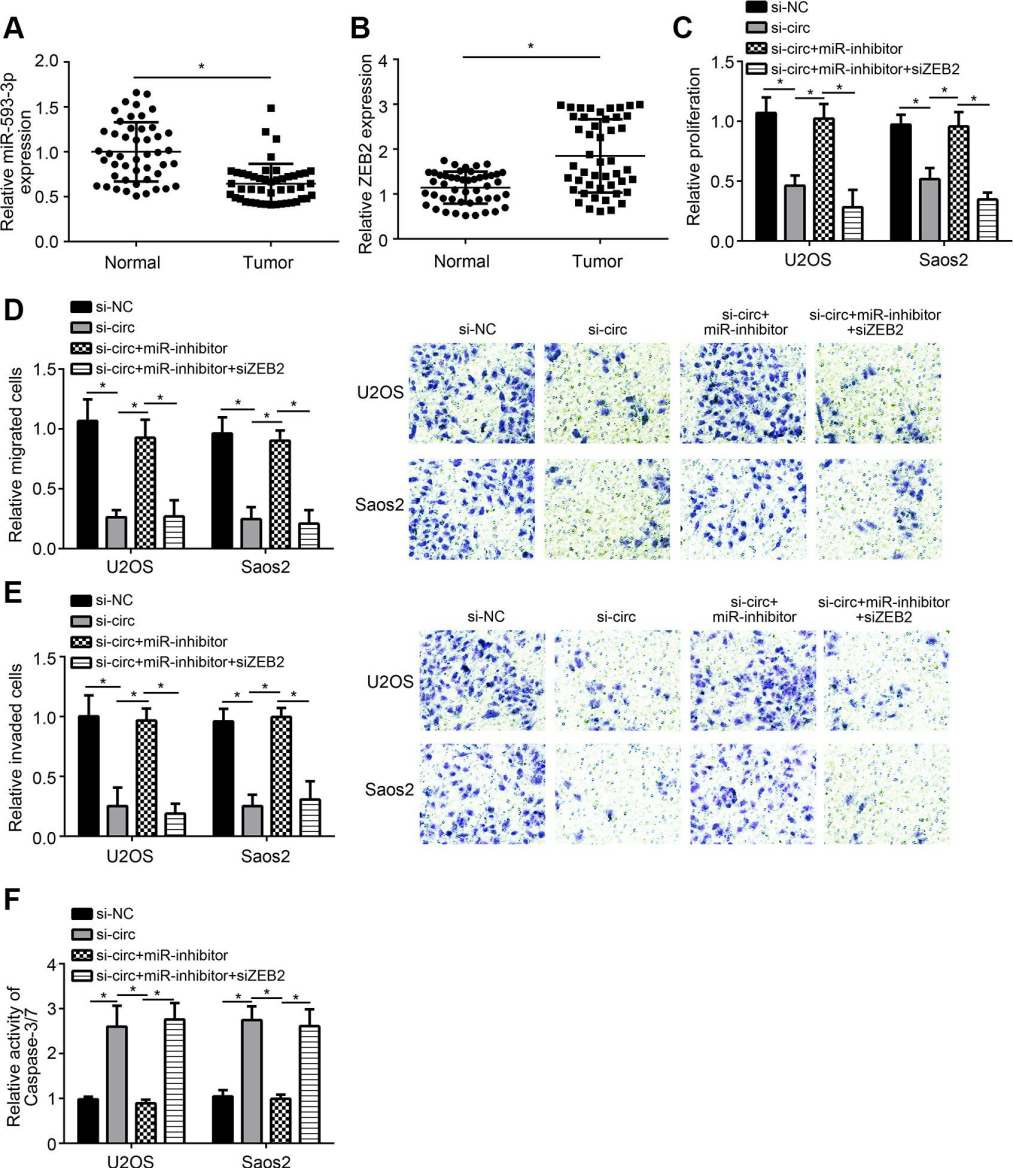
**Effects of hsa_circ_0136666/miR-593-3p/ZEB2 axis on OS progression.** (**A**, **B**) Relative expression of miR-593-3p and ZEB2 in OS tissues. (**C**) Relative proliferation was measured by CCK8 assay. miR-inhibitor: miR-593-3p inhibitor. (**D**, **E**) Migration and invasion were evaluated by Transwell assay. miR-inhibitor: miR-593-3p inhibitor. (**F**) Apoptosis detection via analysis of Caspase-3/7 activity. miR-inhibitor: miR-593-3p inhibitor. *P<0.05 by Student’s t-test. Results from at least three independent experiments were presented as mean ± standard deviation.

We found that miR-593-3p expression was downregulated in OS tissues (Figure 4A). And ZEB2 expression was upregulated in OS tissues (Figure 4B). To confirm whether miR-593-3p/ZEB2 axis regulates OS progression, we performed rescue experiments. Based on CCK8, Transwell and apoptosis detection assays, we found that miR-593-3p inhibition rescued the proliferation, migration, invasion and survival of U2OS and Saos2 cells transfected with hsa_circ_0136666 siRNA (Figure 4C–4F). Moreover, additional silencing of ZEB2 further suppressed proliferation, migration and invasion while inducing apoptosis (Figure 4C–4F). Summarily, hsa_circ_0136666 modulates miR-593-3p/ZEB2 axis to promote OS progression.

### Hsa_circ_0136666/miR-593-3p/ZEB2 axis contributes to OS growth in vivo

**Figure 5:**
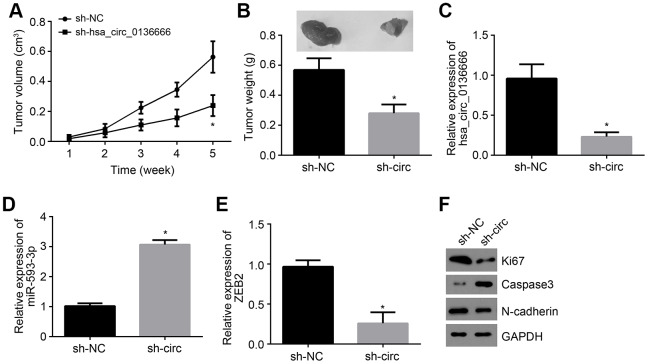
**hsa_circ_0136666/miR-593-3p/ZEB2 axis contributes to OS growth *in vivo*.** (**A**) Tumor size was measured every one week. N=5 for each group. (**B**) Tumor weight was analyzed after 5 weeks post injection. N=5 for each group. (**C**–**E**) Relative expressions of hsa_circ_0136666, miR-593-3p and ZEB2 in tumor tissues were measured by qRT-PCR. (**F**) Protein levels of Ki67, Caspase3 and N-cadherin were detected by Western blotting. *P<0.05 by Student’s t-test. Results from at least three independent experiments were presented as mean ± standard deviation.

To further validate the role of hsa_circ_0136666/miR-593-3p/ZEB2 axis in vivo, animal experiments were performed. Every one week, tumor sizes were examined and 5 weeks later, tumor weight was determined. It was found that hsa_circ_0136666 silencing suppressed tumor growth (Figure 5A, 5B). We also measured the expressions of hsa_circ_0136666, miR-593-3p and ZEB2 in tumor tissues. Results indicated that hsa_circ_0136666 and ZEB2 expressions were decreased in tumor tissues of si-circ group while miR-593-3p level was increased (Figure 5C–5E), indicating that hsa_circ_0136666/miR-593-3p/ZEB2 axis contributes to OS growth *in vivo*. Moreover, we found that hsa_circ_0136666 silencing promoted apoptosis while inhibiting proliferation and invasion (Figure 5F).

## DISCUSSION

How to cure OS is still a major problem for human health. Efforts to search therapeutic targets for OS treatment is still of significant importance. In this study, we elucidated the relationship between hsa_circ_0136666 expression and OS progression. We showed that hsa_circ_0136666 expression was upregulated in OS tissues and predicted poor prognosis. And knockdown of hsa_circ_0136666 significantly suppressed the proliferation, migration and invasion while causing more cell death. We also demonstrated that hsa_circ_0136666 plays its function through sponging miR-593-3p to enhance ZEB2 expression. Our findings suggested hsa_circ_0136666/miR-593-3p/ZEB2 axis may be a new target for OS therapy.

The presence of circRNA was validated 30 years ago. However, study on its function is just emerging. Recent studies have demonstrated circRNAs possess very important biological roles in tumorigenesis, including OS development [15, 16]. For example, hsa_circ_0009910 is reported to promote carcinogenesis of OS via sponging miR-449a [17]. CircRNA hsa_circ_0001564 participates in OS progression via regulating cell proliferation and apoptosis [18]. CircRNA circNASP promotes growth and invasion of OS cells through inhibiting miR-1253 [19]. Hsa_circ_0136666 has been shown to promote colon cancer development and breast cancer progression [13, 14]. Nevertheless, whether hsa_circ_0136666 regulates OS development is unclear. In our study, hsa_circ_0136666 expression was identified to be upregulated in OS tissues. Moreover, overexpression of hsa_circ_0136666 is related to advanced stage and poor prognosis. In addition, knockdown of hsa_circ_0136666 inhibited the proliferation, migration and invasion of OS cells. Thus, our results suggest hsa_circ_0136666 as an oncogene in OS.

We then researched the molecular mechanism. Previously, hsa_circ_0136666 was reported to sponge miRNAs [13, 14]. Consistently, we also identified that hsa_circ_0136666 may be a sponge for miR-593-3p via bioinformatics analysis. We demonstrated their association via luciferase reporter assay. Moreover, we found that miR-593-3p level was increased after hsa_circ_0136666 knockdown in OS cells. MiR-593-3p is a potential tumor suppressor in gastric cancer and lung cancer. Dong et al. reported miR-593-3p inhibited proliferation and metastasis of gastric cancer cells [20]. Han et al. showed that miR-593-3p represses lung cancer growth and migration [21]. However, whether miR-593-3p exerts a similar role in OS remains unclear. In our results, we found that miR-593-3p was downregulated in OS tissues and it suppressed proliferation, migration and invasion of OS cells. Thus, miR-593-3p was a tumor suppressor in OS and acted downstream of hsa_circ_0136666.

Afterwards, we also confirmed the target of miR-593-3p via bioinformatics analysis. We identified ZEB2 and validated the interaction between ZEB2 and miR-593-3p by luciferase reporter assay. The expression of ZEB2 was suppressed by miR-593-3p. Also, we demonstrated that hsa_circ_0136666 knockdown led to ZEB2 downregulation by stimulating miR-593-3p. We then found that ZEB2 was upregulated in OS tissues, implying a potential oncogenic role. ZEB2 is a widely known oncogene in various cancers, including lung cancer [22], breast cancer [10] and OS [23]. In consistent with those findings, we also demonstrated that ZEB2 silencing impaired proliferation, migration and invasion of OS cells, supporting its oncogenic roles.

Taken together, our work demonstrated that hsa_circ_0136666 is overexpressed in OS tissues and promotes tumorigenesis through targeting miR-593-3p/ZEB2 axis, implying hsa_circ_0136666 might be a novel therapeutic target.

## MATERIALS AND METHODS

### Tissue collection

47 pairs of OS tissues and adjacent normal controls were obtained from the First Affiliated Hospital of Soochow University. These tissues were frozen in liquid nitrogen. Our study was approved by the Ethics Committee of the First Affiliated Hospital of Soochow University (20180602035) and written informed consent was achieved from involved patients. Experiments involving human tissues were conducted in accordance with the Declaration of Helsinki.

### Cell culture

hFOB1.19 cell line and human OS cell lines were obtained from Shanghai Academy of Sciences. Cell lines were maintained in DMEM medium (Invitrogen, USA) supplemented with 10% fetal bovine serum (FBS, Gibco, USA).

### Cell transfection

Si-hsa_circ_0136666 (5’-ACAGUCUCUUUGUUGGGCAAT-3’), miR-593-3p mimics (5’-UGUCUCUGCUGGGGUUUCU-3’), miR-593-3p inhibitors (5’-AGAAACCCCAGCAGAGACA-3’) and negative controls were purchased from GenePharma (Shanghai, China). Cell transfection was carried out using Lipofectamine 3000 reagent (Invitrogen) according to the protocols of manufacturer.

### qRT-PCR

Total RNAs were extracted using Trizol reagent (Invitrogen) and qPCR was performed as reported before [24]. Primer sequences were as follows: hsa_circ_0136666 (Forward, 5’-AGGTGCTCACTGTGCTGAAA-3’; Reverse, 5’-CAGATGTTCATTGGGTCCAT-3’); mIR-593-3p (Forward, 5’-AGAATCTGTCAGGCACCAGCC-3’; reverse, 5’-ACAAACCCAGCACCACTCCT-3’), U6 (Forward, 5’-ATACAGAGAAAGTTAGCACGG-3’; reverse, 5’-GGAATGCTTCAAAGAGTTGTG-3’) GAPDH (Forward, 5’-TCAAGATCATCAGCAATGCC-3’; reverse, 5’-CGATACCAAAGTTGTCATGGA-3’).

### Cell counting kit-8 (CCK8)

CCK8 (Dojindo, Tokyo, Japan) was used to test the ability of OS cells. In brief, 2000 cells were seeded into the 96-well plates and cultured for indicated times. Then CCK8 solution was added and incubated for 2 hours. Absorbance at 450 nm was determined using a microplate reader.

### Colony forming assay

1000 cells were seeded into the 6-well plates and cultured for 14 days. The clones were then fixed and stained. Colony numbers were eventually counted.

### Animal study

Animal experiments were approved by the ethic committee of the First Affiliated Hospital of Soochow University (20180602082). U2OS cells were injected into the flank of recipient nude mice (n=5 for each group). At indicated time points, the tumor volumes were measured. And 5 weeks later, tumor weights were determined.

### Transwell assay

Migration and invasion were measured using Transwell assay as previously described [6]. In brief, after 24h of transfection, 2×10^4^ cells per well were seeded into the upper chamber (Corning, NY, USA) with serum-free medium. The lower chamber was filled with 600 μl complete medium. After culture for 24 h, the migrated or invaded cells in the lower chamber was fixed and stained with crystal violet. Cell number was then counted.

### Isolation of nuclear and cytoplasmic RNA

This assay was conducted as previously described [25]. In brief, Nuclear and cytoplasmic RNAs were isolated using a PARIS™ kit (Thermo Fisher Scientific) according to the manufacturer’s instructions. After purification and DNA digestion, RNA was reverse transcribed and used for qPCR.

### Measurement of Caspase3/7 activity

The Caspase-3/7 activities were detected with Apo-ONE homogenous caspase 3/7 activity assay kit (Promega, Madison, WI) according to the manufacturer’s instruction.

### Luciferase reporter assay

The sequences containing miR-593-3p binding site in hsa_circ_0136666 (nucleotides: 200~400) or ZEB2 (nucleotides: 0~200 of the 3’-UTR) were inserted into the pGL3 luciferase vector (Promega). For luciferase reporter assay, the wild-type or mutant luciferase reporter was transfected into the U2OS cells as well as miR-593-3p mimics or negative control. 24 h later, the luciferase activity was determined using the Dual-Luciferase Reporter Assay (Promega, Madison, WI). And the relative firefly luciferase activity was normalized to Renilla activity.

### Data analysis

Data were analyzed by SPSS 22.0 (IBM, USA) and presented as mean ± standard deviation. Statistical differences were calculated using Student’s t-test or One-way ANOVA. Clinical data were analyzed using Kaplan-Meier curve and the log-rank test. *P* < 0.05 was considered significant.

### Data availability statement

All data were included in this manuscript.
